# Electrical stimulation of the sciatic nerve restores inspiratory diaphragm function in mice after spinal cord injury

**DOI:** 10.3389/fncir.2024.1480291

**Published:** 2025-01-22

**Authors:** Ian Walling, Sarah Baumgartner, Mitesh Patel, Steven A. Crone

**Affiliations:** ^1^Neuroscience Graduate Program, University of Cincinnati, College of Medicine, Cincinnati, OH, United States; ^2^Medical Scientist Training Program, University of Cincinnati, College of Medicine, Cincinnati, OH, United States; ^3^Division of Neurosurgery, Cincinnati Children’s Hospital Medical Center, Cincinnati, OH, United States; ^4^Neurobiology Program, University of Cincinnati, College of Arts and Sciences, Cincinnati, OH, United States; ^5^Division of Developmental Biology, Cincinnati Children’s Hospital Medical Center, Cincinnati, OH, United States; ^6^Department of Neurosurgery, University of Cincinnati, College of Medicine, Cincinnati, OH, United States

**Keywords:** spinal cord injury, respiration, nerve stimulation, sciatic nerve, sensory afferent, electromyography, diaphragm

## Abstract

**Introduction:**

Spinal cord injury in the high cervical cord can impair breathing due to disruption of pathways between brainstem respiratory centers and respiratory motor neurons in the spinal cord. Electrical stimulation of limb afferents can increase ventilation in healthy humans and animals, but it is not known if limb afferent stimulation can improve breathing following a cervical injury.

**Methods:**

We stimulated the sciatic nerve while using electromyography to measure diaphragm function in anesthetized mice following a cervical (C2) hemisection spinal cord injury, as well as in uninjured controls. The amplitude and frequency of inspiratory bursts was analyzed over a range of stimulation thresholds.

**Results:**

We show that electrical stimulation (at sufficient current thresholds) of either the left or right sciatic nerve could restore inspiratory activity to the previously paralyzed diaphragm ipsilateral to a C2 hemisection injury at either acute (1 day) or chronic (2 months) stages after injury. We also show that sciatic nerve stimulation can increase the frequency and amplitude of diaphragm inspiratory bursts in uninjured mice.

**Discussion:**

Our findings indicate that therapies targeting limb afferents could potentially be used to improve breathing in patients with cervical spinal cord injury and provide an experimental model to further investigate the neural pathways by which limb afferents can increase respiratory muscle activity.

## Introduction

Respiratory failure is the most common cause of death in people with cervical spinal cord injury (SCI; Berlly and Shem, 2007; Berlowitz et al., 2016). Moreover, impaired breathing or coughing can lead to potentially fatal infections. People living with spinal cord injury that require mechanical ventilation have a significantly decreased lifespan compared to those that do not (Berlly and Shem, 2007; van den Berg et al., 2010; Waddimba et al., 2009). Respiratory deficits in people with cervical SCI are caused by the loss of descending projections from brainstem respiratory regions to respiratory motor neurons below the injury. Many cervical spinal cord injuries are not anatomically complete, rather the spared pathways between the brain and spinal cord are merely insufficient for function (Fouad et al., 2021; Kirshblum et al., 2011; Waring et al., 2010; Waters et al., 1991). Current therapies are limited to mechanical ventilation or diaphragmatic pacing, which do not address the underlying cause of insufficient respiratory drive to respiratory motor neurons (Berlowitz et al., 2016; Hachem et al., 2017). For this reason, these treatments have a limited capacity to respond to changes in activity or oxygen demand. Moreover, long-term mechanical ventilation elevates the risk of pneumonia (Garcia-Leoni et al., 2010). Although pre-clinical studies have shown potential to improve breathing via electrical stimulation of cervical and/or thoracic regions (Dimarco and Kowalski, 2013; Huang et al., 2016; Huang et al., 2022; Kowalski et al., 2019; Malone et al., 2022; Mercier et al., 2017), translation of these findings to humans with spinal cord injury has thus far been limited (DiMarco et al., 2006, 2009; Kandhari et al., 2022; Malone et al., 2021). Thus, new therapies that have the capacity to restore brainstem control of respiration to people with cervical spinal cord injury are needed.

Locomotor and respiratory function are coordinated in healthy people and animals to ensure that respiratory function is appropriate for any activity level (Forster et al., 2012; Forster and Pan, 1988, 1994; Guyenet and Bayliss, 2015; Schottelkotte and Crone, 2022). Passive limb movements in humans can increase ventilation (Bell and Duffin, 2003; Dejours et al., 1959a; Dejours et al., 1959b; Gozal et al., 1996; Noah et al., 2008; Zhuang et al., 2009), likely due to activation of the locomotor central pattern generator circuits by limb proprioceptive afferents. Importantly, coordination of limb and respiratory function is achieved through feedforward mechanisms that anticipate increases in activity and not just feedback mechanisms that rely on changes in blood gasses. For example, activation of the mesencephalic locomotor region can increase breathing concomitant with (or even in the absence of) an increase in locomotion (Herent et al., 2023). An increase in respiratory activity during locomotion is also partially mediated by projections from lumbar spinal cord neurons to the parafacial region of the brainstem important for breathing (Herent et al., 2023; Kanbar et al., 2016; Korsak et al., 2018; Le Gal et al., 2014). For example, in anesthetized animals (e.g., rabbit, dog, and rat), stimulation of hindlimb afferents or the sciatic nerve increases respiratory frequency and amplitude (Haxhiu et al., 1984; Kanbar et al., 2016; Korsak et al., 2018; Mizumura and Kumazawa, 1976; Schiefer et al., 2018). Additional pathways, including intraspinal connections between lumbar and cervical circuits, may also participate in coordination of locomotor and respiratory activity. For example, electrophysiological experiments have demonstrated the presence of dual locomotor and respiratory neurons in the spinal cord (Le Gal et al., 2016). Further, activation of spinal networks can drive phrenic motor neuron bursting even in the absence of bulbospinal projections (Cregg et al., 2017). These findings suggest that activation of locomotor circuits could potentially impact breathing.

Clinical studies have shown that locomotor training can improve pulmonary function in adults living with spinal cord injury (Carvalho et al., 2005; Hormigo et al., 2017; Randelman et al., 2021; Soyupek et al., 2009; Terson de Paleville et al., 2013; Tiftik et al., 2015). Ability based training can also produce dramatic improvements in ventilation in young children with spinal cord injury (Goode-Roberts et al., 2021). Notably, full pulmonary benefits require active walking (e.g., initiated by the patient- with or without electrical stimulation of the spinal cord), as passive movements generated by a robotic device are not sufficient (Jack et al., 2011). Improvements in breathing due to treadmill training are likely mediated at least in part by changes in respiratory circuits, as opposed to just muscular and/or metabolic changes. Evidence for this includes studies showing that patients with SCI have increased locomotor-respiratory coupling during treadmill walking than uninjured controls (Sutor et al., 2022) and that motor training can reduce the variability in minute ventilation normally observed in people living with SCI (Panza et al., 2017). Thus, locomotor activity appears to have a benefit on breathing in people with spinal cord injuries, but the mechanisms are not well understood.

The C2 hemisection (C2Hx) model for SCI has been widely used to investigate plasticity of respiratory circuits after injury in rabbits, rats, mice, cats, and dogs (DiMarco and Kowalski, 2011; Ford et al., 2016; Jensen et al., 2019; Jensen et al., 2024; Porter, 1895; Zholudeva et al., 2017). By surgically lesioning one lateral half of the cord at C2, the diaphragm ipsilateral to injury is paralyzed while the contralateral diaphragm’s inspiratory activity is unimpaired. Increasing chemosensory drive (e.g., paralyzing the functional diaphragm by phrenicotomy) can restore rhythmic inspiratory activity to the ipsilateral diaphragm. Restoration of activity to the previously paralyzed diaphragm is thought to be mediated by spared “crossed phrenic pathways” from the brainstem that cross the midline of the spinal cord below C2 (Goshgarian, 2003). Restoration of function to the ipsilateral diaphragm can also be accomplished by activating glutamatergic propriospinal neurons (Jensen et al., 2024; Satkunendrarajah et al., 2018), which may provide alternative pathways for the brainstem to activate phrenic motor neurons or provide tonic drive that allows phrenic motor neurons to respond to weak crossed phrenic pathways (Jensen et al., 2024). Over time, some degree of spontaneous recovery of diaphragm function may occur, likely due to altered connectivity within circuits below the site of injury (Courtine et al., 2009; Courtine et al., 2008; Formento et al., 2018; Jensen et al., 2024; Lorach et al., 2022; Zholudeva et al., 2017). Thus, the C2Hx model is well-suited to assess methods to improve respiratory function after injury at both acute and chronic time points.

This study tests the hypothesis that activation of locomotor circuits by electrical stimulation of the sciatic nerve can improve diaphragm function after a cervical spinal cord injury. We first show that sciatic nerve stimulation (to activate hindlimb afferents) can increase diaphragm activity in uninjured mice, as has been shown previously in other species. We then show that sciatic nerve stimulation can restore function to the previously paralyzed diaphragm 1 day after a C2Hx injury, when stimulation is applied either ipsilateral or contralateral to injury. Finally, we show that sciatic nerve stimulation is even more effective at chronic time points than acutely following injury. These results indicate that sciatic nerve stimulation has the potential to improve breathing after injury. Further, this model can be used in future studies to identify the circuits and mechanisms by which respiratory motor neurons are activated by sciatic nerve/limb afferent stimulation.

## Methods

### C2 hemisection injury model

All animal procedures were performed using C57BL/6J mice (JAX: 000664) according to the National Institutes of Health guidelines and approved by the Cincinnati Children’s Hospital Medical Center animal care and use committee’s regulations. Surgical lesions were performed on one lateral half of the spinal cord at C2 in adult male mice (60–176 days of age) under 1 L/min 1% isoflurane, as previously described (Jensen et al., 2024). A 2 cm incision was made at midline beginning at the back of the skull and extending to between the scapula of the animal, exposing the trapezius. The trapezius was then cut at midline caudal to rostral using microscissors to expose the paravertebral muscles. Paravertebral muscles were blunt dissected using cotton tipped applicators to expose the posterior skull and cervical vertebrae. Laminectomy of C1 and C2 vertebrae was performed using microscissors to expose the spinal cord. Once exposed, a durotomy of the spinal cord was performed using forceps, then a 30-gauge needle was inserted near the midline at the rostral edge of the C2 vertebra and passed laterally through the tissue to create a left hemisection injury. Up to 5 needle passes were performed until complete injury was achieved, which was determined by observation of lack of chest wall movement on the side ipsilateral to injury. Paravertebral and trapezius muscles were sutured back together following injury, and the incision was closed using dermal adhesive. Following surgery, 5 mg/kg*BW carprofen was administered subcutaneously for analgesia, nails were trimmed to prevent possible injury during grooming, and the animal was placed into a 32°C incubator overnight. Cages were supplied with nutritional gel and a water bottle. 5 mg/kg*BW carprofen was administered the morning after surgery and as needed for continued analgesic care. Diaphragm EMG recordings/ stimulation experiments were performed between 6 and 12 h after carprofen administration (for recordings performed 1 day post injury). Animals also received subcutaneous injections of 1.0 mL saline on the 1^st^ and 2^nd^ post-operative day. Animals which underwent 2 month post-injury recordings received weekly nail trimming to prevent injury when grooming.

### Diaphragm electromyography and sciatic nerve stimulation

Bilateral diaphragm EMG recording was performed under isoflurane in cohorts of uninjured animals, animals 1 day following C2Hx surgery, and 2 months following C2Hx surgery. Recordings were performed under 0.8–1% isoflurane, 1 L/min O2 flow anesthesia with body temperature maintained at 36°C using a heating pad with thermal probe, and animals placed in the prone position with forelimbs and tail secured using surgical tape to stabilize the animal. This anesthetic level was found to prevent any response to a foot pinch (prior to stimulation) and tail pinch (post sciatic nerve cut). Animals initially underwent exposure of the sciatic nerve bilaterally, with 2 cm incisions placed over the back legs of the animal and blunt dissection of the biceps femoris and gluteus maximus to expose the sciatic nerve. A cuff electrode (Microprobes microcuff electrode-consisting of 3 rings of 100um platinum wire spaced 1 mm apart, in an insulated cuff with a 0.5 mm inner diameter and 3 mm at each end outside of wires) was primed with sterile saline and placed around the exposed nerve. The nerve and cuff were covered with mineral oil to prevent drying out of the tissue. A 5 cm incision in the skin at the base of the rib cage exposed the oblique muscles, and 2 cm lateral incisions of the obliques on each side of the spine exposed the caudal surface of the diaphragm through the peritoneal cavity. Two sets of bipolar electrodes (each consisting of 2 NEE-3 needle electrodes (CWE Inc.) taped together with 1.5 mm spacing between electrode tips) connected to a BMA-400 AC/DC bioamplifier were inserted into the left and right side of the diaphragm, with grounded electrodes inserted into the cut external obliques. EMG signals were acquired and analyzed using Spike2 data analysis software (CED). A 30 Hz-10 kHz band pass filter was applied by the amplifier during signal acquisition.

Electrical stimulation was delivered using a WPI Model A365 constant current stimulus isolator. Stimulation was controlled using the Spike2 program, with key activated triggers. The threshold of stimulation required to elicit a motor response (T) was determined by stimulating over a range of currents (1, 5, 10, 15 and 20 μA) until a motor response was observed in the hind paw. The average current required to elicit activation of dorsiflexion/plantarflexion in the ipsilateral paw was found to be 9.3 +/− 1.9uA in healthy mice for both left and right sciatic nerve stimulation. For C2Hx animals at 1 day post injury, the average stimulation to elicit a motor response was 8.1uA+/−2.6 for the left (ipsilateral to injury) and 7.8uA+/− 2.7 for the right (contralateral to injury) sciatic nerve. For C2Hx animals at 2 months post injury, the average stimulation to elicit a motor response was 8.9uA+/− 2.2 for the left and 7.9uA+/−2.7 for the right sciatic nerve. We did not use currents above 200 μA in order to prevent potential activation of high threshold c-fiber nociceptive afferents (Sdrulla et al., 2015). Animals which did not exhibit motor response to stimulation (e.g., due to damage to the sciatic nerve during cuff placement) with both nerve cuffs were excluded from the study. T was assessed independently for both left and right sciatic nerves. The sciatic nerve was cut distal to the cuff following assessment of T to prevent limb movement on subsequent stimulations. Next, animals underwent a brief 15 s nasal occlusion to ensure that maximal chemosensory drive results in rhythmic activation of the paralyzed diaphragm, indicating that crossed phrenic pathways remain intact (Jensen et al., 2024; Mantilla et al., 2011). After nasal occlusion, a 10 min baseline was recorded. Following baseline, animals underwent sciatic nerve stimulation. Stimulation was delivered for 1 min in 20 Hz, 1 ms bipolar pulses, followed by a 1 min period of no stimulation. Except as noted, the same animal underwent consecutive stimulation on the left and right sciatic nerves at 1×, 2×, 5×, 10×, 15×, and 20× T. One cohort of animals underwent a different stimulation paradigm 1 day following C2Hx, with the goal of testing the effects of time under anesthesia on response to stimulation. For these experiments, stimulation was delivered at 10T current only at 1, 10, and 20 min following baseline. Out of a total of 40 animals, we excluded 2 due to incomplete paralysis of the ipsilateral diaphragm at 24 h post injury, 5 for failure to respond to nasal occlusion with rhythmic activity in the paralyzed diaphragm, 3 animals were excluded due to damage to the sciatic nerves during cuff, and 3 due to damage to the diaphragm from the leads during placement and/or animal movement. Additionally, 5 animals had stimulation delivered unilaterally to one nerve only, due to damage of the other sciatic nerve during cuff placement.

**Figure 1:**
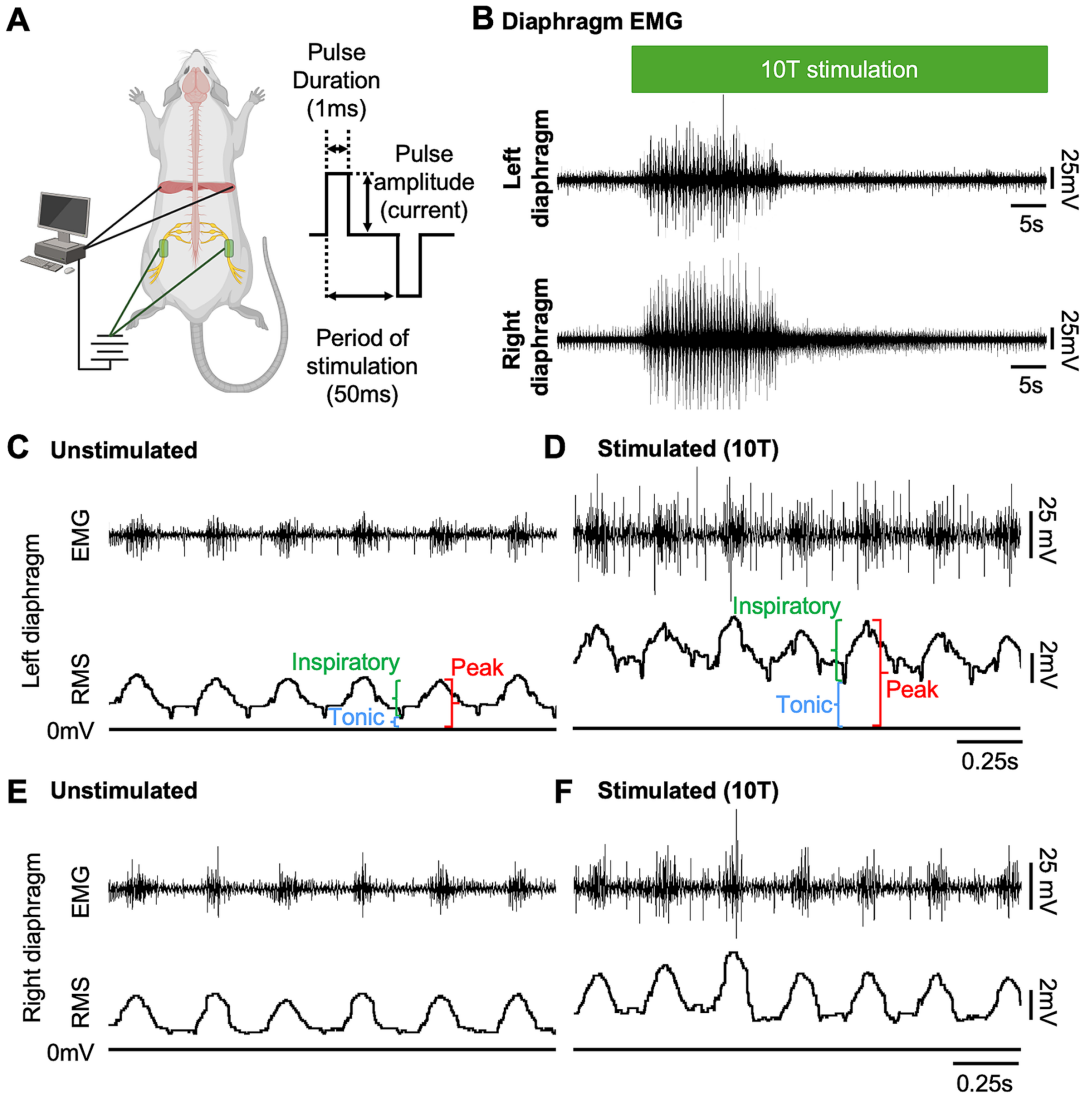
Sciatic nerve stimulation increases diaphragm EMG activity in uninjured mice. **(A)** Diagram of experimental setup showing approximate locations of peripheral nerve cuff electrodes used for sciatic nerve stimulation and EMG electrodes for recording diaphragm activity in an anesthetized mouse (left). Stimulation parameters (bipolar square waveform with 50 ms period and 1 ms pulse duration) used for stimulus are illustrated at right. **(B)** Examples of left and right diaphragm EMG traces from an uninjured mouse prior to and during sciatic nerve stimulation. Stimulation (green bar) was delivered at 10 times the current required to elicit a motor response (10T) for 60 s. **(C–F)** Diaphragm EMG (top) and root mean square (RMS) of EMG signal for the left **(C,D)** and right **(E,F)** diaphragm prior to **(C,E)** and following **(D,F)** stimulation at 10T. Peak, tonic (during expiration), and inspiratory (=peak—tonic) amplitudes were measured for each breath from the RMS signal as shown.

### Electromyography analysis

Acquired EMG signals were processed using a Finite Impulse Response (FIR) digital high-pass 60 Hz filter to remove DC components of the recording as well as movement artifacts. In channels where ECG activity was greater than inspiratory diaphragm activity, the ECG signal was reduced using “ECGDelete02” script (CED) in Spike2. This script subtracts an averaged QRS complex (ECG contamination) from the EMG signal based on a rolling average of 30 previous ECG complexes. Subsequently the root mean square (RMS) was calculated for the channel using a rolling 50 ms window (Jensen et al., 2024; Mantilla et al., 2014; Mantilla and Sieck, 2013). The RMS signal was used to measure the peak amplitude (highest RMS signal for each inspiratory period) and the tonic amplitude (the minimum RMS amplitude during non-inspiratory intervals) for selected 10 s intervals prior to stimulation, during peak stimulation, during the last 10 s of stimulation, and 10 s immediately after stimulation ceased. Two animals (one at 1 day and one at 2 months post-injury) contained a low frequency/high amplitude artifact (consistent with a movement artifact) during stimulation at 10 and 15T which was not removed by our previously described signal processing. For these animals, only regions of the trace that did not contain the low-frequency/high amplitude artifact were analyzed. Periods of inspiration were determined based on the right (contralateral to injury) diaphragm RMS signal. The inspiratory amplitude was calculated by subtracting the tonic amplitude from the inspiratory amplitude for each breath in the analyzed area. Inspiratory frequency was measured prior to (approximately 30s before stimulation) and during stimulation (the same 10s intervals used for determining peak amplitude) by counting the number of inspiratory peaks over a 10s interval and dividing by 10.

### Assessing extent of injury by histology

Upon completion of recordings, animals were terminally anesthetized with 0.2 ml pentobarbital, perfused with 6 ml cold phosphate buffered saline (1x PBS), followed by 1 ml*BW 4% PFA in 1xPB, the spinal cord and brainstem dissected and post-fixed in 4% PFA in 1x PB solution for 2 h. Tissue was then washed in 1x PBS overnight at 4°C to remove excess PFA, then cryoprotected in 30% sucrose for between 8 and 24 h. Cervical spinal cord (C1 to C4) was embedded in OCT and kept in a -80C freezer until sectioned (transverse) at 60 μM using a cryostat. Sections were collected sequentially on slides. Eriochrome cyanin stain was performed by processing tissue slides in the following solutions sequentially: 2× 30 min. Xylene, 3 min. Each of 100% ethanol, 95% ethanol, 70% ethanol, 50% ethanol, and 2 min. in dH20. Eriochrome stain was made by mixing 12 ml of 1% FeCl_3_ + 2.7% HCl with 240 ml of 0.2% Eriochrome cyanin in 0.5% H_2_SO_4_ and bringing the total volume to 300 ml using dH_2_O. Slides were stained with Eriochrome stain for 10 min, then excess stain was washed off using 2 dips in milliQ H_2_O. After washing excess stain, a 30 s differentiation step was performed in a solution of 0.5% aqueous NH_4_OH in dH_2_O, followed by washing off excess solution by dipping in dH_2_O twice. Slides was then air dried overnight in a chemical fume hood. The following day, slides were placed into xylene for 10 min, and allowed to dry for 20 min prior to cover slipping with Permount. Permount was allowed to harden overnight prior to further handling. Tissue was imaged using a Nikon Ni-E upright motorized microscope using a 4x objective lens. The section with maximal damage was assessed empirically, then used to calculate the extent of injury (Jensen et al., 2024; Mantilla et al., 2014). Using NIS-Elements, the spared white and grey matter of the cord were outlined and the area was calculated (TotalSparedTissueArea). To estimate the total area of the cord, the area of the uninjured half of the cord (HemicordArea) was doubled. Percent spared tissue area was calculated using the following calculation:

$$\%\mathrm{Spared}=\frac{\mathrm{TotalSparedTissueArea}}{2\;x\;\mathrm{HemicordArea}}x\;100$$

### Statistical analysis

Repeated measures statistics were used to analyze the changes in EMG amplitude and respiratory rate between stimulated and unstimulated conditions. ANOVAs were utilized where data collected was found to be normally distributed, while a Friedman’s F-test (for non-parametric data) was used when the distribution was not found to be normal (see Tables 1–3). For each group of animals, peak amplitude was compared at pre-stimulation baseline versus the 1, 2, 5, 10, and 15T stimulation conditions for each side of the diaphragm (left and right) and each sciatic nerve stimulated (left and right; see Tables 1–3). Tonic and inspiratory amplitude were analyzed similarly (see Tables 1–3). Dunn’s test (which controls for multiple comparisons) was used for post-hoc analysis to compare pre-stimulation baseline to each intensity of stimulation. A multivariate repeated measures ANOVA was used to analyze the changes in frequency during stimulation, comparing the frequency immediately prior to a given stimulation versus the frequency during stimulation for each of 1, 2, 5, 10 and 15T. Comparisons were made between the pre- and post-stimulated values for each stimulation tested, and the Bonferroni correction was used to control for multiple comparisons when using an ANOVA. In all tests, a *p*-value below 0.05 was considered statistically significant. *P* and *F* values for each test are given in Tables 1–3. Power analyses (*α* = 0.05, power = 80%) determined that we had a sufficient number of animals to detect a 49% change from baseline in uninjured animals, 63% change from baseline in the diaphragm ipsilateral to injury in the one-day post C2Hx group, and an 81% change from baseline in the diaphragm ipsilateral to injury in the two-months post C2Hx group.

**Table 1:** Uninjured mice statistical analyses.

Source data	Property	Conditions	*N* = mice	Test	*F* value/Friedman Statistic	*p* value
Figure 2A	Peak EMG	Left stim, left diaphragm	7	Friedman	18.27	**0.0026**
Figure 2C	Tonic EMG		7	Friedman	20.63	**0.0010**
Figure 2E	Inspiratory EMG		7	Friedman	14.02	**0.0155**
Figure 2A	Peak EMG	Right stim, left diaphragm	7	Friedman	8.143	0.1485
Figure 2C	Tonic EMG		7	Friedman	9.204	0.1012
Figure 2E	Inspiratory EMG		7	Friedman	5.612	0.3458
Figure 2B	Peak EMG	Left stim, right diaphragm	7	Friedman	17.86	**0.0031**
Figure 2D	Tonic EMG		7	Friedman	17.04	**0.004**
Figure 2F	Inspiratory EMG		7	Friedman	10.67	0.0583
Figure 2B	Peak EMG	Right stim, right diaphragm	7	Friedman	6.592	0.2528
Figure 2D	Tonic EMG		7	Friedman	10.18	0.0702
Figure 2F	Inspiratory EMG		7	Friedman	5.694	0.3372
Figure 3A	Respiratory Rate	Left stim, right diaphragm	7	2 way ANOVA	3.638	**0.0109**
Figure 3B	Respiratory Rate	Right stim, right diaphragm	7	2 way ANOVA	3.636	**0.0109**

*p values less than 0.05 are bold.*

**Table 2:** One day post C2Hx mice statistical analyses.

Source data	Property	Conditions	*N* = mice	Test	*F* value/Friedman Statistic	*p* value
Figure 5A	Peak EMG	Left stim, ipsi diaphragm	8	Friedman	29.07	**<0.0001**
Figure 5C	Tonic EMG		8	Friedman	19.98	**0.0013**
Figure 5E	Inspiratory EMG		8	Friedman	28.23	**<0.0001**
Figure 5A	Peak EMG	Right stim, ipsi diaphragm	7	Friedman	28.48	**<0.0001**
Figure 5C	Tonic EMG		7	Friedman	1.185	0.9463
Figure 5E	Inspiratory EMG		7	Friedman	21.50	**0.0007**
Figure 5B	Peak EMG	Left stim, contra diaphragm	8	Friedman	23.21	**0.0003**
Figure 5D	Tonic EMG		8	Friedman	20.05	**0.0012**
Figure 5F	Inspiratory EMG		8	Friedman	23.14	**0.0003**
Figure 5B	Peak EMG	Right stim, contra diaphragm	7	Friedman	23.41	**0.0003**
Figure 5D	Tonic EMG		7	Friedman	4.118	0.5326
Figure 5F	Inspiratory EMG		7	Friedman	18.18	**0.0027**
Figure 6A	Respiratory Rate	Left stim, contra diaphragm	8	2 way ANOVA	7.538	**<0.0001**
Figure 6B	Respiratory Rate	Right stim, contra diaphragm	7	2 way ANOVA	4.395	**0.0040**
Figure 7A	Inspiratory EMG	1 day post C2Hx, left stim, 10T current, ipsi diaphragm	5	ANOVA	15.69	**0.0112**
Figure 7B	Inspiratory EMG	1 day post C2Hx, right stim, 10T current, ipsi diaphragm	5	ANOVA	10.66	**0.0278**

*p values less than 0.05 are bold.*

**Table 3:** Two months post C2Hx mice statistical analyses.

Source data	Property	Conditions	*N* = mice	Test	*F* value/Friedman Statistic	*p* value
Figure 8E	Peak EMG	Left stim, ipsi diaphragm	9	Friedman	25.13	**0.0001**
Figure 8G	Tonic EMG		9	Friedman	12.17	**0.0325**
Figure 8I	Inspiratory EMG		9	Friedman	26.40	**<0.0001**
Figure 8E	Peak EMG	Right stim, ipsi diaphragm	7	Friedman	26.84	**<0.0001**
Figure 8G	Tonic EMG		7	Friedman	9.857	0.0794
Figure 8I	Inspiratory EMG		7	Friedman	25.69	**0.0001**
Figure 8F	Peak EMG	Left stim, contra diaphragm	9	Friedman	14.84	**0.0111**
Figure 8H	Tonic EMG		9	Friedman	12.17	**0.0325**
Figure 8J	Inspiratory EMG		9	Friedman	9.508	0.0904
Figure 8F	Peak EMG	Right stim, contra diaphragm	7	Friedman	5.122	0.4011
Figure 8H	Tonic EMG		7	Friedman	8.878	0.1140
Figure 8J	Inspiratory EMG		7	Friedman	6.755	0.2395
Figure 9B	Respiratory Rate	Left stim, contra diaphragm	9	2 way ANOVA	6.242	**0.0002**
Figure 9A	Respiratory Rate	Right stim, contra diaphragm	7	2 way ANOVA	2.827	**0.0330**

*p values less than 0.05 are bold.*

Figures 1A, 4A were created with BioRender.com.

**Figure 2:**
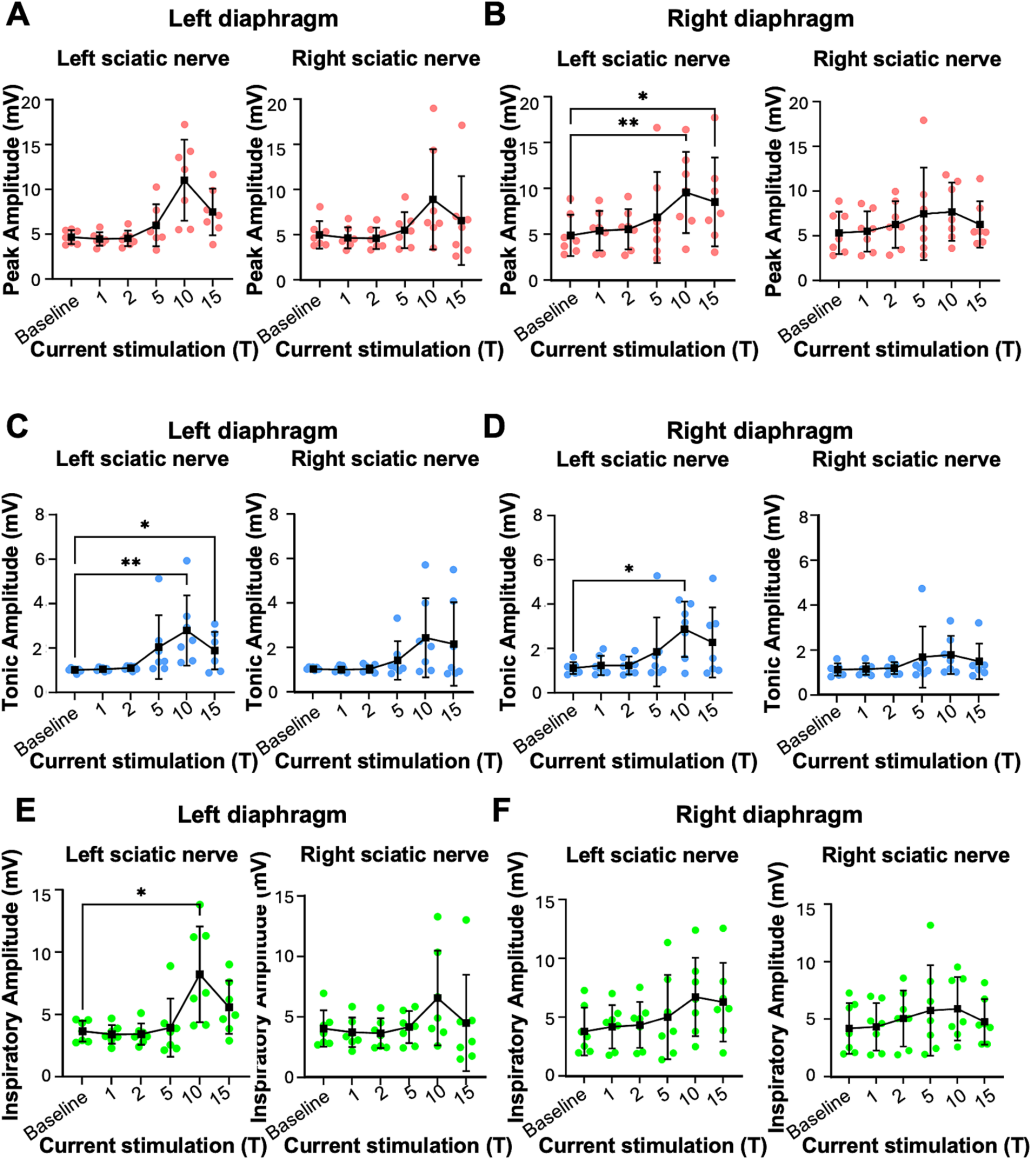
Peak, tonic and inspiratory amplitude of diaphragm EMG in uninjured mice. Anesthetized mice were instrumented with cuff electrodes to stimulate the left and right sciatic nerve as well as electrodes to measure left and right sides of the diaphragm. One sciatic nerve was stimulated at a time (alternating between left and right), sequentially at currents corresponding to 1, 2, 5, 10, 15 times threshold (T). **(A,B)** Peak amplitude in the left **(A)** and right **(B)** diaphragm during stimulation of the left and right sciatic nerves at thresholds from 1-15T. Each red data point represents the average peak amplitude over a 10s window from one mouse during the peak of stimulation or baseline (pre-stimulation). The average for all animals is indicated by black squares. **(C,D)** Tonic amplitude for the left **(C)** and right **(D)** diaphragm during stimulation of the left and right sciatic nerves at 1–15T. Each blue dot represents the average tonic amplitude for one animal whereas the average for all animals is indicated by black squares. **(E,F)** Inspiratory amplitude in the left **(E)** and right **(F)** diaphragm during stimulation at 1–15T or baseline. Each green dot represents the average inspiratory amplitude for one animal whereas the average for all animals is indicated by black squares. Error bars = standard deviation. Statistical significance was determined using the Friedman *F*-test comparing stimulated values with the unstimulated baseline, with post-hoc Dunn’s test. *p value<0.05, **p value<0.01, *N* = 7 mice for left sciatic stimulation and 7 for right sciatic stimulation.

**Figure 3:**
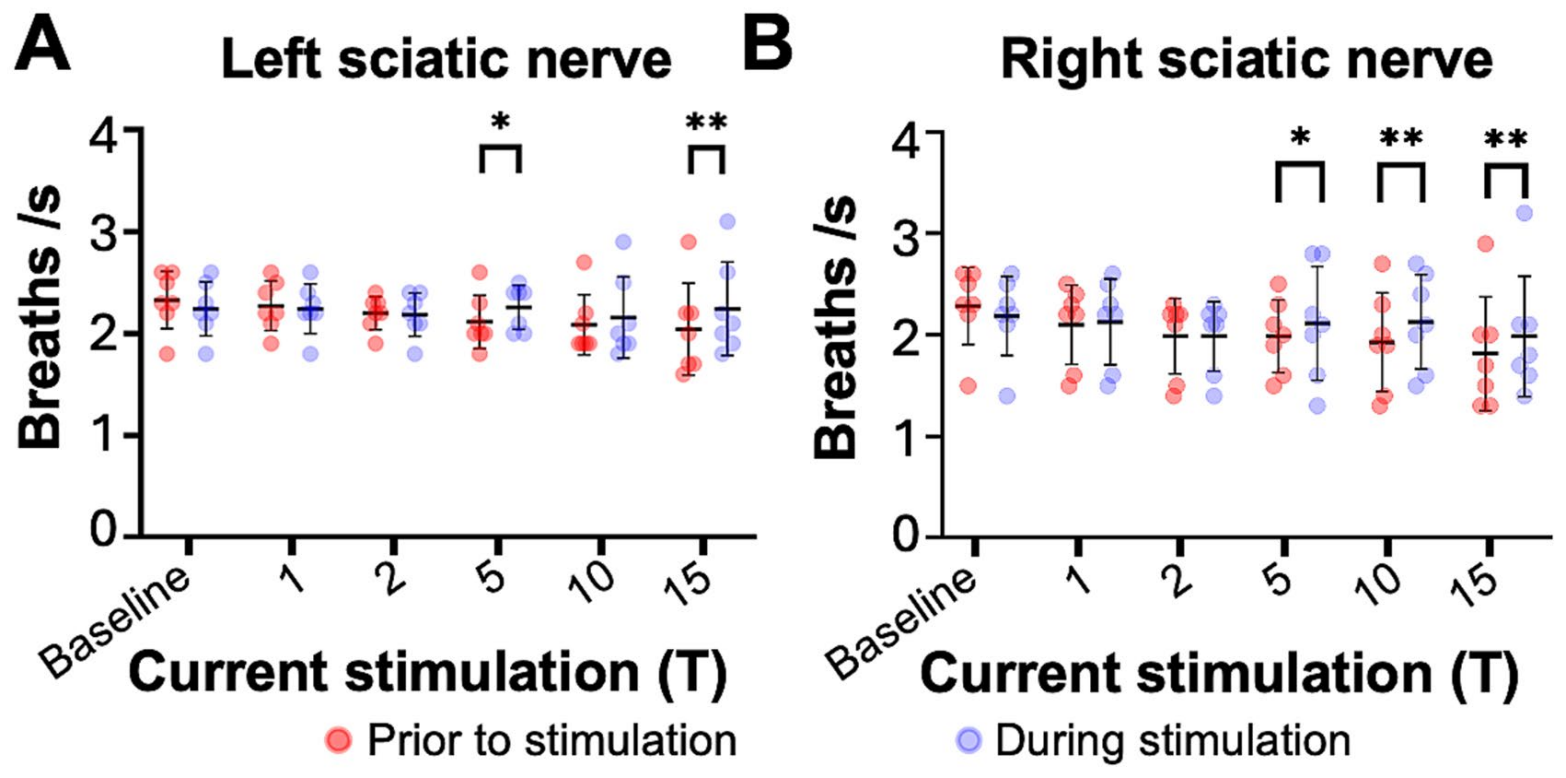
Sciatic nerve stimulation increases respiratory frequency in uninjured mice. Respiratory frequency was calculated from the inspiratory RMS signal by counting the number of inspiratory peaks in a 10s interval just prior to (red) and during (blue) left **(A)** and right **(B)** sciatic nerve stimulation at 1-15T, during the same periods analyzed in Figure 4. Baseline recordings include two 10s intervals (red and blue) within the last minute prior to any electrical stimulation. The average frequency for the group is represented by a black line, while the error bars show the standard deviation of the group. Statistical comparisons were made using a 2-way repeated measures ANOVA (stimulation intensity and before versus during stimulation), with post-hoc testing using Bonferroni correction. * *p* value <0.05, ** *p* value<0.01, *N* = 7 mice for left sciatic stimulation and 7 mice for right sciatic stimulation.

## Results

### Electrical stimulation of the sciatic nerve increases diaphragm activity in healthy mice

We tested whether electrical stimulation of hindlimb afferents could increase diaphragm activity in mice, as has previously been shown in other species (Haxhiu et al., 1984; Kanbar et al., 2016; Korsak et al., 2018; Mizumura and Kumazawa, 1976; Schiefer et al., 2018). In anesthetized, freely breathing adult mice, a cuff electrode was applied to the sciatic nerve on both the left and right hindlimbs. The sciatic nerve contains sensory and motor axons innervating a significant portion of the skin and muscles of the leg and foot. Needle electrodes were inserted into each side of the diaphragm to record electromyograph (EMG) activity (Figure 1A). Electrical pulses (1 ms pulse width, 20 Hz, 1 min train; Figure 1A) were delivered over a range of currents to determine the threshold sufficient to elicit a motor response (T) in the hind paw distal to each cuff electrode. The sciatic nerve distal to each cuff was cut to eliminate further hindlimb responses. We stimulated one sciatic nerve at a time (alternating between left and right), sequentially at currents corresponding to 1, 2, 5, 10, 15, and 20 times threshold (1–20T). However, since 20T often resulted in strong thoracic movements such that the diaphragm was occasionally damaged by the inserted electrodes, we eliminated this time point from our analyses. No movement of the head was noted during these high threshold stimulations. We elected to perform all the experiments in this study in non-ventilated, non-vagotomized mice to preserve their ability to respond to changes in blood gasses and simulate what might be observed in healthy or injured humans undergoing stimulation. As expected, electrical stimulation of the sciatic nerve at sufficient thresholds could increase the amplitude of inspiratory diaphragm activity during stimulation (Figure 1B). Diaphragm activity is increased both ipsilateral and contralateral to the stimulated nerve. Prolonged stimulation led to an attenuation of these effects by the end of the 60s period of stimulation (Figure 1B). To analyze in more detail the effects of sciatic nerve stimulation on diaphragm activity, we compared the EMG activity on both sides of the diaphragm during a 10s baseline period (prior to any stimulation) as well as a 10s period containing the peak activation of the diaphragm during stimulation (starting between 4 and 25 s after the onset of stimulation). The average duration of the experiment was 10 min (baseline) plus 28+/−9 min for stimulations. For each period, we analyzed the root mean square (RMS) of the diaphragm activity and used this to measure the peak amplitude (during inspiration) and tonic amplitude (during expiration) of the diaphragm EMG signal. To calculate the inspiratory amplitude, we subtracted the tonic from the peak amplitude for each inspiratory burst (Figure 1C). Examples of diaphragm EMG and RMS traces for the left and right diaphragm prior to and during stimulation are shown in Figures 1C–F. We measured peak diaphragm activity for each condition (left/right nerve stimulation and left/right diaphragm EMG; Figures 2A,B). We observed an increase in peak amplitude with left sciatic nerve stimulation that was statistically significant (Table 1). Tonic activity was also elevated significantly by stimulation of the left sciatic nerve (Figures 2C,D; Table 1). The inspiratory amplitude also showed a tendency towards increased amplitude upon stimulation but was only statistically significant for the left diaphragm following left sciatic nerve stimulation (Figures 2E,F; Table 1). Thus, stimulation of the sciatic nerve at high thresholds (10–15T) can elicit an increase in peak, tonic and inspiratory amplitude, but the degree of change from baseline (normal inspiratory activity) is variable in uninjured mice.

**Figure 4:**
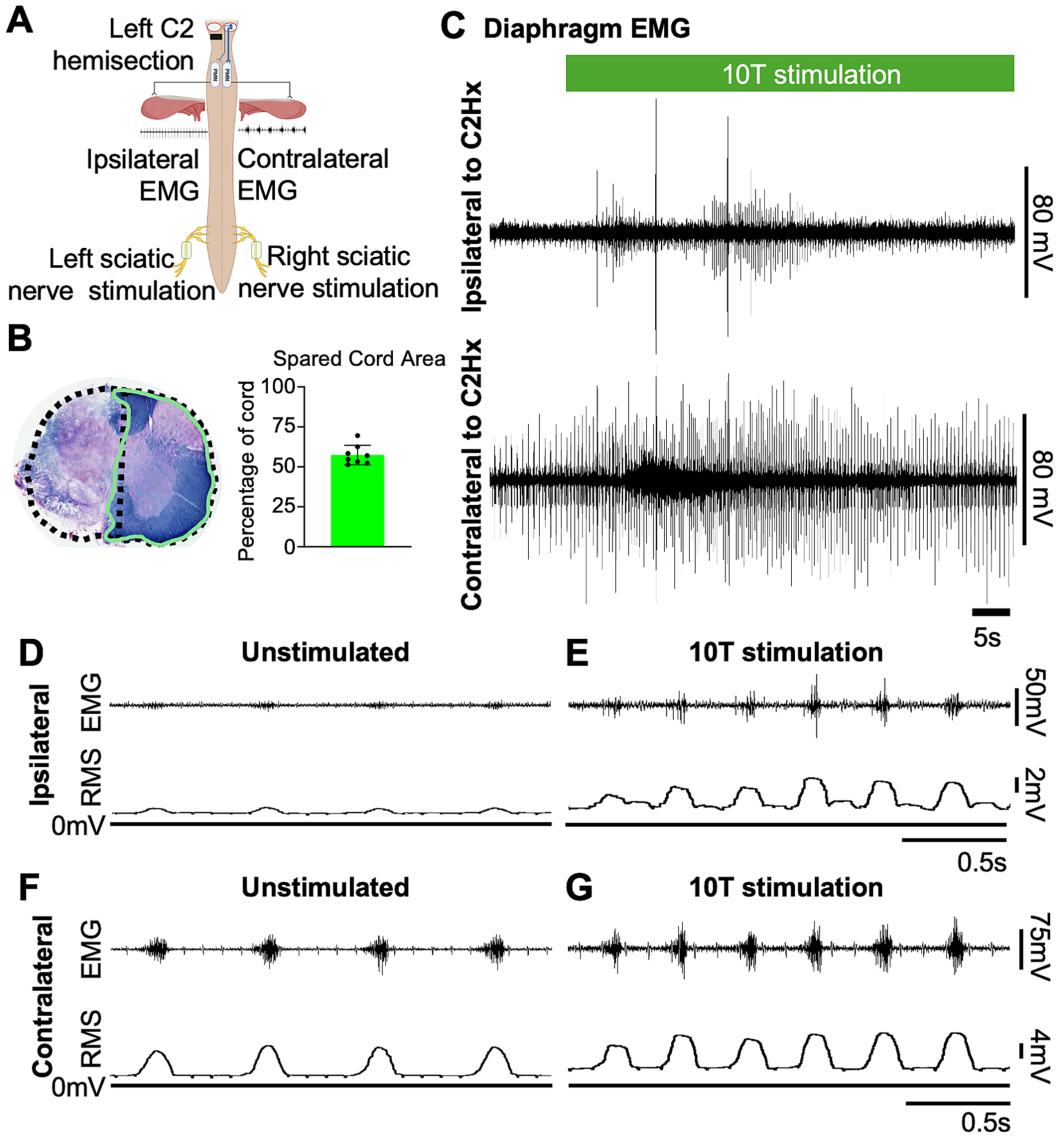
Sciatic nerve stimulation restores inspiratory activity in the paralyzed diaphragm one day after a C2 hemisection injury. **(A)** Diagram showing the approximate locations of a left C2 hemisection injury (performed 1 day prior to recording) and the relative position of recording electrodes (ipsilateral and contralateral diaphragm EMG) and cuff electrodes (left and right) for sciatic nerve stimulation. **(B)** Eriochrome staining was performed on cervical tissue sections to label white matter of each cord at the site of greatest injury. Stained tissue was imaged, and extent of injury was assessed by measuring the area of spared tissue (green outline). The presumed area of the intact cord was estimated by doubling the area of the hemicord on the uninjured side (dashed line) in order to calculate the % of spared tissue. Bar graph shows the average spared cord area as a % of the whole cord, with dots representing individual mice (*n* = 8 mice). **(C)** Examples of diaphragm EMG traces recorded from the ipsilateral (top) and contralateral (bottom) side relative to a C2Hx injury prior to and during sciatic nerve stimulation. Stimulation (green bar) was delivered at 10 times the current required to elicit a motor response (10T) for 60 s. **(D,E)** Diaphragm EMG and RMS recorded ipsilateral to the C2Hx injury prior to **(D)** and during **(E)** stimulation of the left sciatic nerve at 10T. **(F,G)** Diaphragm EMG and RMS recorded contralateral to the C2Hx injury prior to **(F)** and during **(G)** stimulation of the left sciatic nerve at 10T.

We next examined changes in respiratory rate following sciatic nerve stimulation. The number of inspiratory peaks in the RMS signal were counted for a 10s window just prior to stimulation and for a 10s window during stimulation at the time of peak diaphragm activation. There was a significant increase in breathing frequency during left and right sciatic nerve stimulation compared to baseline (Table 1). This increase was seen at 5–15T stimulation, but not at lower currents (Figures 3A,B). The elevated respiratory rate persisted through the last 10s of stimulation (Table 4). Thus, our results confirm that sciatic nerve stimulation can increase both the frequency and amplitude of diaphragm activity in uninjured anesthetized mice.

**Figure 5:**
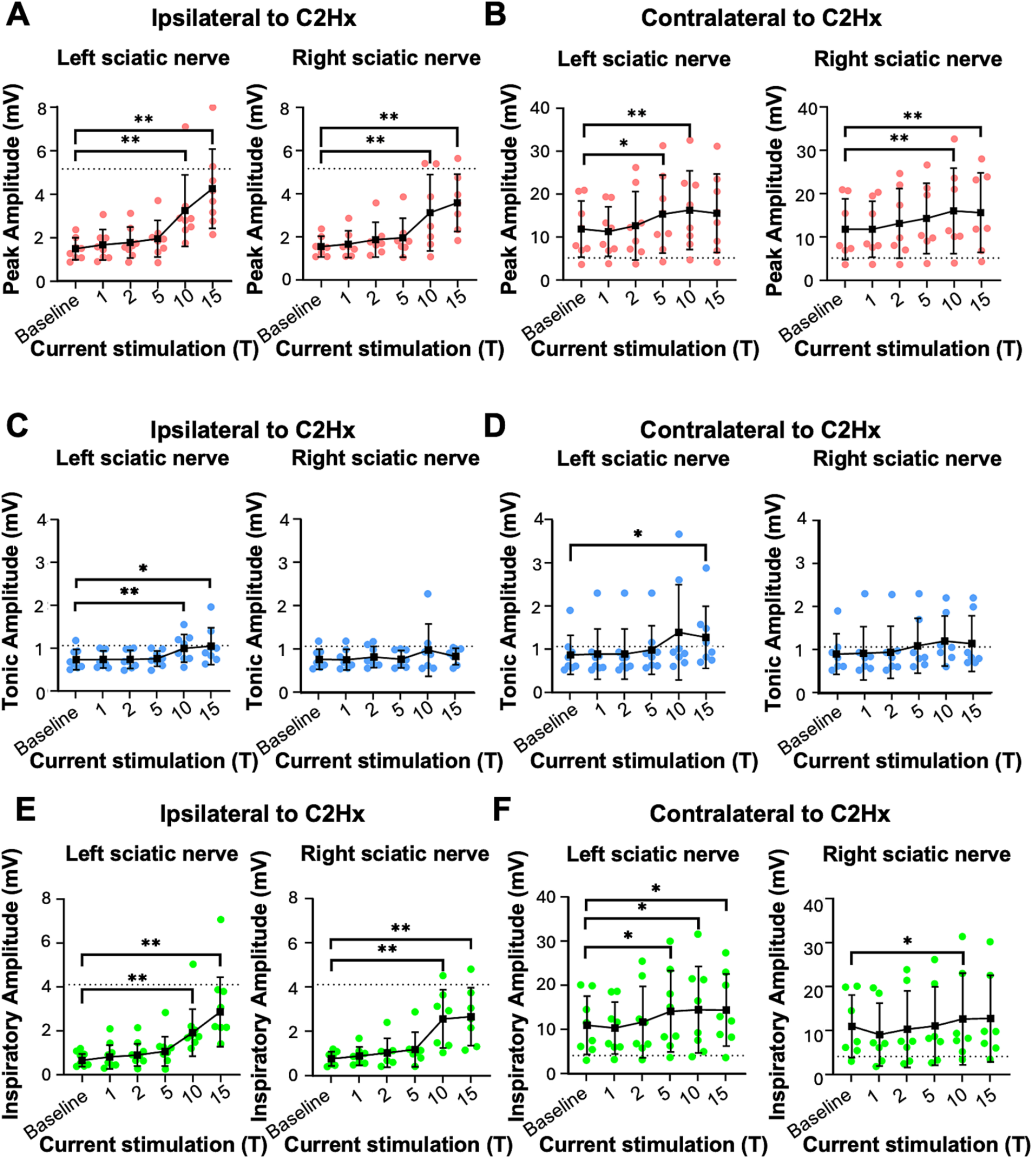
Peak, tonic, and inspiratory amplitude of diaphragm EMG one day after C2Hx injury. Anesthetized mice 1 day following a C2Hx injury were instrumented with cuff electrodes to stimulate the left and right sciatic nerve as well as electrodes to measure left and right sides of the diaphragm. One sciatic nerve was stimulated at a time (alternating between left and right), sequentially at currents corresponding to 1, 2, 5, 10, 15 times threshold (T). **(A,B)** Peak amplitude in the left **(A)** and right **(B)** diaphragm during stimulation of the left and right sciatic nerves at thresholds from 1 to 15T. Each red data point represents the average peak amplitude over a 10s window from one mouse during the peak of stimulation or baseline (pre-stimulation). The average for all animals is indicated by black squares. **(C,D)** Tonic EMG amplitude for the left **(C)** and right **(D)** diaphragm during stimulation of the left and right sciatic nerves. Each blue dot represents the average tonic amplitude for one animal whereas the average for all animals is indicated by black squares. **(E,F)** Inspiratory EMG amplitude in the left **(E)** and right **(F)** diaphragm during stimulation at 1–15T or baseline. Each green dot represents the average inspiratory amplitude for one animal whereas the average for all animals is indicated by black squares. The dotted lines in **(A–F)** indicate the average peak, tonic, or inspiratory amplitude of uninjured mice at baseline (from Figure 4). Error bars = standard deviation. Statistical significance was determined using the Friedman F-test comparing stimulated values with the unstimulated baseline, with *post-hoc* Dunn’s test. * *p* value<0.05, ** *p* value<0.01, *N* = 8 mice (left sciatic nerve), 7 mice (right sciatic nerve).

**Table 4:** Effects of stimulation on respiratory frequency.

Stimulated nerve	Current	Prior to stim (breaths/s)			Peak (breaths/s)			Last 10 s of stim (breaths/s)			*N* (Mice)
Injured											
Left sciatic	0	2.33	±	0.28							7
	5t	2.11	±	0.26	2.26	±	0.21	2.23	±	0.27	
	10t	2.10	±	0.31	2.16	±	0.40	2.13	±	0.39	
	15t	2.04	±	0.46	2.24	±	0.46	2.11	±	0.47	
Right sciatic	0	2.28	±	0.38							7
	5t	1.99	±	0.36	2.11	±	0.56	2.13	±	0.54	
	10t	1.92	±	0.49	2.13	±	0.47	2.07	±	0.52	
	15t	1.81	±	0.56	1.99	±	0.59	1.89	±	0.64	
One day post injury											
Left sciatic	0	1.33	±	0.30							8
	5t	1.19	±	0.26	1.38	±	0.28	1.25	±	0.31	
	10t	1.16	±	0.26	1.55	±	0.67	1.33	±	0.35	
	15t	1.15	±	0.32	1.54	±	0.42	1.13	±	0.32	
Right sciatic	0	1.30	±	0.32							7
	5t	1.24	±	0.28	1.37	±	0.23	1.21	±	0.20	
	10t	1.09	±	0.27	1.34	±	0.33	1.13	±	0.25	
	15t	1.04	±	0.36	1.25	±	0.50	1.07	±	0.27	
Two months post injury											
Left sciatic	0	1.81	±	0.38							9
	5t	1.82	±	0.34	1.97	±	0.38	1.72	±	0.34	
	10t	1.78	±	0.35	1.94	±	0.28	1.61	±	0.32	
	15t	1.66	±	0.25	1.98	±	0.33	1.60	±	0.45	
Right sciatic	0	1.83	±	0.43							7
	5t	1.64	±	0.44	1.69	±	0.38	1.66	±	0.35	
	10t	1.57	±	0.36	1.71	±	0.33	1.57	±	0.49	
	15t	1.47	±	0.40	1.73	±	0.40	1.49	±	0.37	

All values are average breaths per second ± standard deviation.

### Electrical stimulation of the sciatic nerve restores inspiratory activity to the paralyzed diaphragm after a C2 hemisection injury

Having established that sciatic nerve stimulation increases diaphragm output in healthy mice, we tested the effects of stimulation on diaphragm function after a cervical spinal cord injury. We utilized a C2 hemisection injury (C2Hx) in which the left half of the spinal cord is surgically transected at C2, paralyzing the diaphragm ipsilateral to the injury while the diaphragm contralateral to injury continues to contract during inspiration and maintain ventilation (Figure 4A). Post-hoc evaluation of the extent of injury was performed by harvesting the cervical cords, sectioning, and staining with eriochrome cyanin to detect myelin. We quantified the extent of spared tissue in the section containing the most damage and expressed it as a percentage of the expected cross-sectional area of the intact cord (Figure 4B). The spared cord area averaged 57+/− 6% (mean +/− SD), with a range of 51 to 69%.

One day following injury, animals were anesthetized, nerve cuff electrodes applied to the left (ipsilateral to injury) and right (contralateral to injury) sciatic nerves, and EMG electrodes inserted into the left and right sides of the diaphragm. Previous studies demonstrated that at 1 day after injury there is no significant spontaneous recovery but one can reliably activate the paralyzed diaphragm using maximal chemosensory drive (i.e., the crossed phrenic phenomenon; Jensen et al., 2024; Minor et al., 2006) or by chemogenetic activation of propriospinal neurons (Jensen et al., 2024). Prior to any electrical stimulation, we measured inspiratory amplitude from the RMS of the diaphragm EMG as described for uninjured animals, using the contralateral diaphragm to assess periods of inspiration. As expected, minimal inspiratory amplitude was observed in the left (ipsilateral to injury) diaphragm of C2Hx animals (0.7 +/− 0.3 mV) compared to uninjured mice (3.5 +/− 1.1 mV). Further, the inspiratory amplitude of the contralateral diaphragm (11.0 +/− 6.6 mV) was increased in C2Hx mice compared to uninjured mice (3.2 +/− 1.6 mV), likely compensating for the paralyzed diaphragm as found in previous studies (Jensen et al., 2024; Minor et al., 2006).

To assess the effects of electrical stimulation of the sciatic nerve on diaphragm activity in C2Hx injured mice, we stimulated one sciatic nerve at a time (alternating between left and right), sequentially at currents corresponding to 1, 2, 5, 10, 15, and 20 times threshold (1–20T), as previously done for uninjured mice. The average duration of the experiment was 10 min (baseline) plus 22+/−4 min for stimulations. We excluded 20T stimulation from our analyses due to the potential for diaphragm damage in some animals. Stimulations above 5T typically resulted in an increase in diaphragm EMG peak amplitude ipsilateral to injury as well as an increase in respiratory frequency (Figure 4C). In contrast to healthy mice that all exhibited an attenuation of the increase in peak amplitude over 60s of stimulation, the majority of C2Hx mice showed a persistent increase in peak amplitude (6/8 mice) and frequency (6/8 mice) throughout the 60s of 10T stimulation. An example of the increase in diaphragm activity during stimulation at 10T compared to the unstimulated condition is shown in Figures 4D–G. Note that stimulation restores inspiratory bursting to the previously paralyzed diaphragm that is synchronous with inspiratory activity of the diaphragm contralateral to injury. We quantified the peak, tonic and inspiratory diaphragm amplitude prior to stimulation as well as during 10s of peak activity during stimulation for each condition (left/right sciatic nerve stimulation, diaphragm EMG ipsilateral/contralateral to injury, 0–15T stimulation). Table 2 describes the statistical tests performed and resulting *F*- and *P*-values. Peak amplitude was significantly elevated in the diaphragm ipsilateral to injury with both left and right sciatic nerve stimulation (10 and 15T; Figure 5A and Table 2). There was also a small (but statistically significant) increase in peak amplitude of the contralateral (not paralyzed) diaphragm during stimulation (Figure 5B). Tonic activity was significantly increased during stimulation of the left sciatic nerve, but not the right sciatic nerve (Figures 5C,D; Table 2). The inspiratory amplitude of the diaphragm ipsilateral to injury was significantly increased by electrical stimulation of either the left (ipsilateral to injury) or right (contralateral to injury) sciatic nerve at thresholds of 10T and 15T, but not at lower thresholds (Figure 5E). The inspiratory amplitude of the contralateral diaphragm was also significantly increased by stimulation (Figure 5F; Table 2). Our results show that electrical stimulation of either sciatic nerve can restore rhythmic inspiratory activity to the previously paralyzed diaphragm after a C2Hx injury.

**Figure 6:**
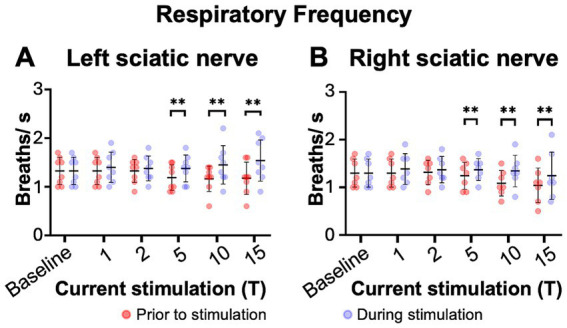
Sciatic nerve stimulation increases respiratory frequency in C2 hemisection injured mice 1 day post injury. Respiratory frequency was calculated from the inspiratory RMS signal of the diaphragm contralateral to injury by counting the number of inspiratory peaks in a 10s interval just prior to (red) and during (blue) left **(A)** or right **(B)** sciatic nerve stimulation at 1–15T, during the same periods analyzed in Figure 6. Baseline recordings include two 10s intervals (red and blue) within the last minute prior to any electrical stimulation. The average frequency for the group is represented by a black line, while the error bars show the standard deviation of the group. Statistical comparisons were made using a 2-way repeated measures ANOVA (stimulation intensity and before versus during stimulation), with post-hoc testing using Bonferroni correction. ** *p* value<0.01, *N* = 8 mice (left sciatic nerve), 7 mice (right sciatic nerve).

The effects of electrical stimulation on respiratory frequency in C2Hx injured animals was measured by comparing the respiratory frequency (measured from the diaphragm RMS contralateral to injury) for a 10s window just prior to stimulation to a 10s window during the peak of stimulation, as described previously for uninjured mice. Stimulation at 5, 10, and 15T resulted in significant increases in respiratory rate for both left (Figure 6A) and right (Figure 6B) sciatic nerve stimulation. An elevated respiratory rate persisted through the last 10 s of stimulation (Table 4). Our results show that electrical stimulation of the sciatic nerve can increase both the frequency and amplitude of diaphragm activity after a C2Hx injury.

**Figure 7:**
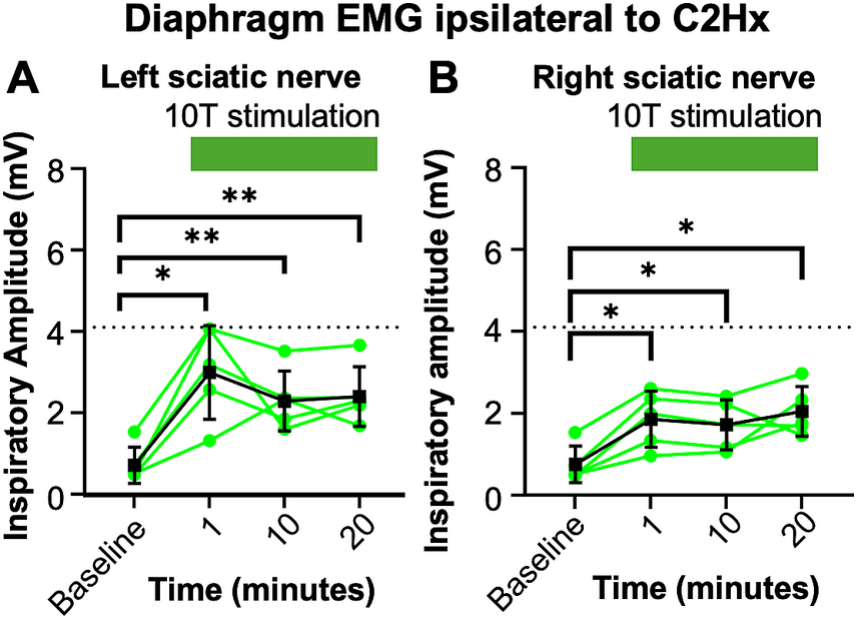
Consistent increase in inspiratory amplitude with repeated 10T stimulation of the sciatic nerve. One day after a C2Hx, left and right diaphragm EMG were recorded prior to nerve stimulation (baseline) or 1, 10, or 20 min after baseline during 10T stimulation of the left **(A)** or right **(B)** sciatic nerve. The inspiratory amplitude was calculated from a 10 s window during the peak response to stimulation or a 10s window prior to stimulation (baseline). Individual animals are represented with green dots, while the group average is shown with black squares. Statistical comparisons were performed using with a repeated measures ANOVA with all timepoints compared to each other, with post-hoc testing using Bonferroni correction. * *p* value <0.05, ** *p* value <0.01, *n* = 5 mice. No statistically significant differences were detected between the 1, 10, and 20 min stimulation timepoints using Bonferroni correction.

We observed a decrease in respiratory frequency prior to stimulation over the course of the experimental protocol (Figure 6), which we hypothesized could be due to a “run-down” effect of anesthesia. Thus, we assessed the potential effect that prolonged anesthesia could have on the ability to activate the diaphragm by sciatic nerve stimulation at a single current threshold (10T). We performed C2Hx injuries and the following day applied sciatic nerve stimulation and recorded diaphragm EMG as previously described. After an initial 10 min baseline period (similar to our previous experiments), animals were stimulated at 10T at 1 min, 10 min, and 20 min time points. The 20 min time point corresponds to the period during which 10T stimulations would have occurred in our previous experiments on healthy and C2Hx animals. Stimulation at 10T consistently produced an increase in inspiratory amplitude in the diaphragm ipsilateral to injury at 1, 10, and 20 min compared to pre-stimulation (Figure 7; Table 2). However, there was not a statistically significant difference in inspiratory amplitude between the 1, 10, and 20 min time points following either left or right sciatic nerve stimulation (Figure 7). Further, no significant difference was observed between inspiratory amplitude following 10T left sciatic nerve stimulation at 1 min in this group of animals (3.0 +/− 1.2 mV) and during the 10T stimulation in the group of animals shown in Figure 5 (2.0 +/− 1.3 mV, Student’s *T*-test, *p* = 0.1770). A similar lack of significance was noted following 10T stimulation of the right sciatic nerve at 1 min (1.8 +/− 0.69 mV) and during 10T stimulation in the group of animals shown in Figure 5 (2.6 +/− 1.3 mV, Student’s *T*-test *p* = 0.2820). Eriochrome staining showed that this group experienced a comparable amount of spared tissue (51 +/− 2% of the cord) following injury as the prior C2Hx injury group (Figure 4; *p* = 0.39). These results demonstrate that 10T stimulation elicits a consistent increase in diaphragm inspiratory amplitude within the time frame of our experiments.

### Inspiratory bursting can be restored to the paralyzed diaphragm by sciatic nerve stimulation 2 months following a C2 hemisection injury

We next tested the ability of sciatic nerve stimulation to restore diaphragm function in mice at chronic stages (2 months) after a C2Hx injury. A cohort of mice underwent a C2Hx injury followed by 2 months of recovery time. Using the same nerve stimulation and diaphragm EMG recording preparation described previously, we stimulated one sciatic nerve at a time (alternating between left and right), sequentially at currents corresponding to 1, 2, 5, 10, 15 and 20 times threshold (1 T-20 T). As with prior experiments, we excluded 20 T stimulations from our analyses due to the potential for diaphragm damage. Prior to stimulation, we observed significant spontaneous recovery in 1 out of 10 mice, so we did not include that animal in our analysis of the effects of nerve stimulation. The remaining 9 animals experienced little spontaneous recovery and the baseline (pre-stimulation) inspiratory amplitude was not significantly different from the 1 day post-C2Hx cohort of animals for the diaphragm ipsilateral to injury (1 day post injury 0.73 +/− 0.33 mV, 2 month post injury 0.79 +/− 0.49 mV, Student’s *T*-test *p* = 0.7863) or for the diaphragm contralateral to injury (1 day post injury 11.0 +/− 6.6 mV, 2 month post injury 9.0 +/− 5.3 mV, Student’s *T*-test, *p* = 0.5030). The extent of injury analysis showed comparable spared tissue area between groups (57 +/− 6% 1 day cohort; 55 +/− 9% 2 month cohort, *p* > 0.99). The average duration of the experiment was 10 min (baseline) plus 23+/−5 min for stimulations.

**Figure 8:**
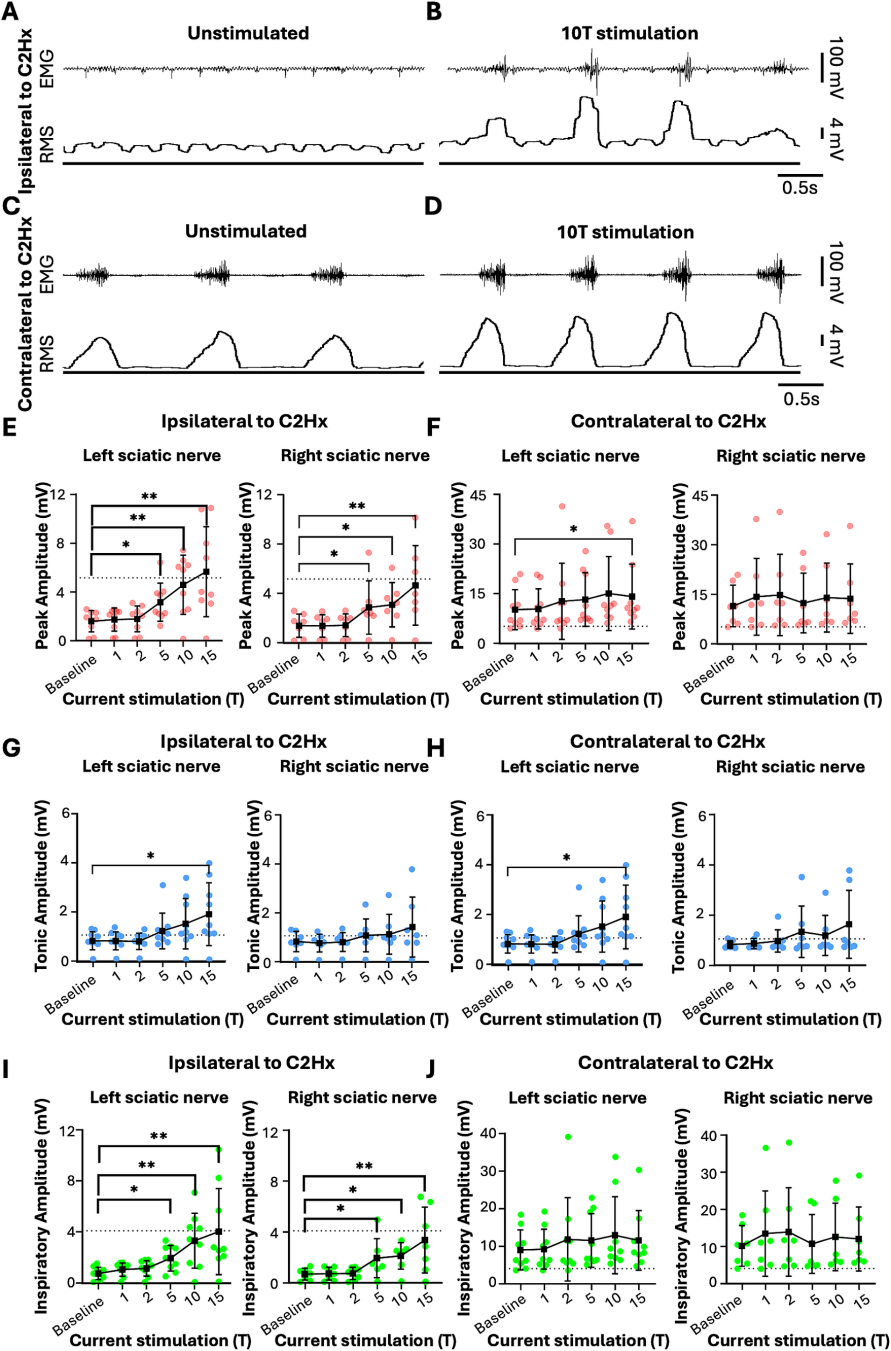
Sciatic nerve stimulation can restore rhythmic inspiratory EMG activity to the paralyzed diaphragm 2 months following C2 hemisection injury. Anesthetized mice 2 months following a C2Hx injury were instrumented with cuff electrodes to stimulate the left and right sciatic nerve as well as electrodes to measure left and right sides of the diaphragm. One sciatic nerve was stimulated at a time (alternating between left and right), sequentially at currents corresponding to 1, 2, 5, 10, 15 times threshold (T). **(A,B)** Peak amplitude in the left **(A)** and right **(B)** diaphragm during stimulation of the left and right sciatic nerves at thresholds from 1 to 15T. Each red data point represents the average peak amplitude over a 10s window from one mouse during the peak of stimulation or baseline (pre-stimulation). The average for all animals is indicated by black squares. **(C,D)** Tonic EMG amplitude for the left **(C)** and right **(D)** diaphragm during stimulation of the left and right sciatic nerves. Each blue dot represents the average tonic amplitude for one animal whereas the average for all animals is indicated by black squares. **(E,F)** Inspiratory amplitude in the left **(E)** and right **(F)** diaphragm during stimulation at 1–15T or baseline. Each green dot represents the average inspiratory amplitude for one animal whereas the average for all animals is indicated by black squares. The dotted lines in **(A–F)** indicate the average peak, tonic, or inspiratory amplitude of uninjured mice at baseline (from Figure 4). Error bars = standard deviation. Statistical significance was determined using Friedman *F*-test comparing stimulated values with the unstimulated baseline, with post-hoc Dunn’s test. * *p* value<0.05, ** *p* value<0.01* *p* value<0.05, ** *p* value<0.01, *N* = 9 mice (left sciatic nerve), 7 mice (right sciatic nerve).

As for animals 1 day after injury, stimulation of the sciatic nerve at 10T could produce rhythmic inspiratory activity in the previously paralyzed diaphragm 2 months following a C2Hx injury (Figures 8A–D). Moreover, peak amplitude was significantly increased in the paralyzed diaphragm at stimulations between 5 and 15T (Figure 8E and Table 3). Thus, at chronic stages, sciatic nerve stimulation could increase diaphragm activity at even lower thresholds (5T) than was typically observed 1 day after injury. The effect of stimulation on the diaphragm contralateral to injury (not paralyzed) was only statistically significant for stimulation of the left sciatic nerve at 15T (Figure 8F). Similar to 1 day after injury, sciatic nerve stimulation could increase tonic diaphragm activity in both the paralyzed and contralateral diaphragm following left sciatic nerve stimulation (Figures 8G,H; Table 3).

**Figure 9:**
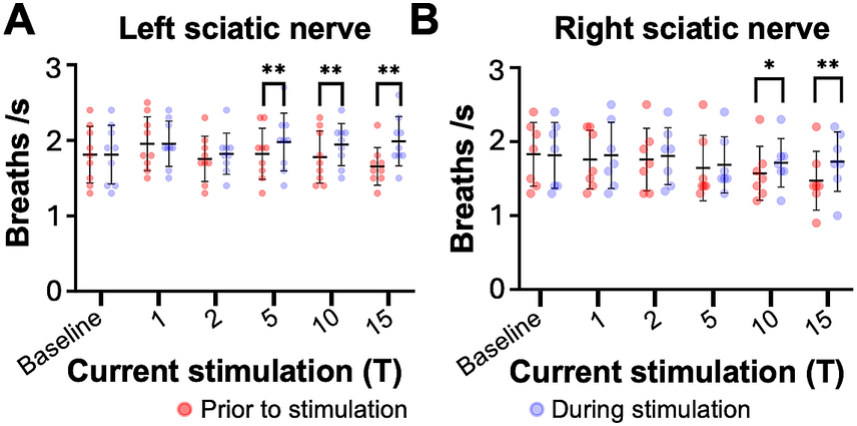
Sciatic nerve stimulation increases respiratory frequency in C2 hemisection injured mice 2 months post injury. Respiratory frequency was calculated from the inspiratory RMS signal of the diaphragm contralateral to injury by counting the number of inspiratory peaks in a 10s interval just prior to (red) and during (blue) left **(A)** or right **(B)** sciatic nerve stimulation at 1–15T, during the same periods analyzed in Figure 8. Baseline recordings include two 10s intervals (red and blue) within the last minute prior to any electrical stimulation. The average frequency for the group is represented by a black line, while the error bars show the standard deviation of the group. Statistical comparisons were made using a 2-way repeated measures ANOVA (stimulation intensity and before versus during stimulation), with post-hoc testing using Bonferroni correction. * *p* value<0.05, **p value<0.01, *N* = 9 mice (left sciatic nerve), 7 mice (right sciatic nerve).

Similar to the peak amplitude, we found that stimulation of the left or right sciatic nerve could increase the inspiratory amplitude of the diaphragm ipsilateral to injury at thresholds between 5 and 15T (Figure 8I; Table 3). In fact, stimulation at the highest thresholds was able to restore peak inspiratory diaphragm activity to levels comparable to uninjured mice. The inspiratory amplitude of the diaphragm contralateral to injury was not consistently increased by stimulation, although some individual animals showed a response (Figure 8J; Table 3). Following the withdrawal of stimulation (the 10s immediately following stimulation), inspiratory amplitude returned to pre-stimulation levels. For example, the inspiratory amplitude increased from 1.5 +/− 0.93 mV prior to left sciatic stimulation, to 3.4 +/− 2.4 mV during stimulation at 10T, and returned to 1.6 +/− 1.9 mV after stimulation (Friedman’s *F* test, *F* = 8.00, *p* = 0.0476 overall, pre to peak stimulation *p* = 0.0368, pre to post stimulation *p* > 0.9999). A similar trend was observed following right sciatic nerve stimulation where the inspiratory amplitude increased from 1.3 +/− 0.83 mV pre-stimulation to 2.1 +/− 1.0 mV during 10T stimulation, then returning to 1.3 +/− 0.70 mV following stimulation (Friedman’s *F* test, *F* = 6.22, p = 0.0476 overall, pre to peak stimulation *p* = 0.0151, pre to post stimulation *p* > 0.9999). As with animals 1 day following injury, stimulation at 5-15T increased respiratory frequency (Figure 9; Table 3). The increase in frequency declined near the end of the 60s stimulation period (Table 4). Thus, at chronic stages, even lower stimulation thresholds are required to elicit significant increases in inspiratory diaphragm activity ipsilateral to a C2Hx compared to 1 day after injury.

## Discussion

In this study, we report the effects of electrical stimulation of the sciatic nerve on diaphragm activity in both healthy and spinal cord injured mice. We found that electrical stimulation can increase diaphragm activity in uninjured mice, as has been observed in other animal models. Further, we found that sciatic nerve stimulation at sufficient thresholds could restore inspiratory activity to the paralyzed diaphragm after a C2 hemisection injury at both acute (1 day) and chronic (2 month) time points after injury. Our findings suggest that limb afferent stimulation could potentially improve respiratory function in people with cervical spinal cord injury.

This study is the first to examine the effects of sciatic nerve stimulation on diaphragm activity in a mouse model. Using electromyography, we found an increase in both the inspiratory frequency and inspiratory amplitude of the diaphragm following sciatic nerve stimulation at current thresholds approximately 10 times larger than required to elicit limb movements. The current thresholds are comparable to findings in dogs (Haxhiu et al., 1984), where diaphragm recruitment began to be seen at about 10 times the threshold to activate A-alpha (proprioceptive) fibers. Previous studies in uninjured animals, including dogs, rats and rabbits, demonstrated that limb nerve stimulation could increase the frequency and amplitude of ventilation (Haxhiu et al., 1984; Kanbar et al., 2016; Korsak et al., 2018; Mizumura and Kumazawa, 1976; Schiefer et al., 2018; Zhuang et al., 2009), and that this was dependent upon intact afferents leading to the cord but not intact motor axons distal to stimulation (Schiefer et al., 2018; Zhuang et al., 2009). Respiratory output has been measured in these studies using airway flow (via pneumotach), respiratory pressure (via transducer), or diaphragm EMG, with similar outcomes to those observed in our study, in that an increase in respiratory rate and inspiratory effort is observed rapidly with onset of stimulation. In addition to an increase in inspiratory amplitude, we also observed a small, but in some cases statistically significant, increase in tonic (expiratory) activity in the diaphragm during electrical stimulation. These changes were observed in both healthy and injured mice. This observation suggests that sciatic nerve stimulation impacts not only the inspiratory rhythm generator in the brainstem, but also non-inspiratory neurons. These non-inspiratory neurons could include expiratory neurons in the brainstem (e.g., parafacial respiratory group) as well as spinal or reticulospinal neurons that may provide additional drive to respiratory motor neurons (Herent et al., 2023; Jensen et al., 2019; Jensen et al., 2024; Korsak et al., 2018; Romer et al., 2017; Satkunendrarajah et al., 2018). Additional studies are necessary to identify the contributions of specific pathways mediating increases in tonic and/or inspiratory amplitude during stimulation.

In healthy mice, over the course of 60 s of stimulation, we typically observed a 20–30 s interval of elevated respiratory activity, followed by a period of attenuation in the increase in amplitude and frequency of respiratory activity, which would be consistent with a hyperventilatory response caused by stimulation of respiratory activity without a concomitant increase in metabolism. Healthy humans undergoing passive limb movements also show an attenuation of the increase in ventilation over time but maintain ventilation at levels higher than at rest (Bell and Duffin, 2003; Dejours et al., 1959a; Dejours et al., 1959b; Gozal et al., 1996; Noah et al., 2008). Intriguingly, in mice that had undergone a C2 hemisection activation was more persistent, with most mice showing sustained increases in amplitude and frequency throughout the 60s stimulation. This change could potentially be a result of a reduced capacity for hyperventilation due to the injury. Our findings indicate that limb afferent-respiratory responses are conserved in the mouse, allowing investigators to make us of the robust genetic tools available in this model to further dissect the circuits and mechanisms responsible.

To our knowledge, this study is the first to assess whether hindlimb afferent stimulation can improve diaphragm function following a C2 hemisection injury. Acutely after injury (1 day), we observed restoration of inspiratory diaphragm EMG activity in the diaphragm ipsilateral to injury in phase with the contralateral diaphragm with stimulation between 10 and 15T. Thus, nerve stimulation produced inspiratory activity in the diaphragm that previously had minimal detectable EMG signal (i.e., was paralyzed). Nerve stimulation could also increase inspiratory amplitude of the contralateral (functional) diaphragm. Importantly, we observed similar diaphragm activation following stimulation of either the left (ipsilateral to injury) or right (contralateral to injury) sciatic nerves. Since most of the ascending pathways are disrupted on one side of the cord following a C2Hx injury, this observation indicates that both ipsilateral and contralateral pathways ascending to the brain and/or intraspinal pathways contribute to diaphragm activation. The observation that sciatic nerve stimulation increases respiratory frequency and not just amplitude indicates that ascending pathways to the brainstem are intact even after a C2Hx injury, as the respiratory rate is controlled by brainstem nuclei (Del Negro et al., 2018; Jensen et al., 2019). Prior studies have indicated that both spinobulbar and intraspinal pathways could contribute to exercise hyperpnea (Forster et al., 2012; Forster and Pan, 1988, 1994; Guyenet and Bayliss, 2015; Herent et al., 2023; Kanbar et al., 2016), and thus it is possible that multiple pathways may play a role. A limitation of this study is that we do not know precisely which types of afferents contribute to the increase in diaphragm activity upon stimulation. Prior studies in uninjured animals (Haxhiu et al., 1984; Mizumura and Kumazawa, 1976) have indicated that small polymodal afferents responding to mechanical, chemical, and noxious stimuli are particularly important for respiratory responses. Our results indicating that high thresholds are required to elicit a respiratory response is consistent with this hypothesis. Although we failed to observe pain-related responses to a foot or tail pinch prior to or subsequent to electrical stimulation (confirming anesthesia), this does not rule out the possibility that stimulation activates pain-related pathways to elicit a change in breathing. We also cannot rule out a role for proprioceptive afferents in activation of respiratory muscles. For example, others have shown that activation of these afferents by vibration can protect infants from apneas of prematurity (Kesavan et al., 2016). Although we limited our stimulations to 200 μA or below with the goal of limiting activation of nociceptive C-fibers, we cannot rule out activation of low threshold nociceptive afferents in our experiments. However, our observation that contralateral stimulation was able to increase respiratory activity suggests that the spinothalamic tract for nociception (Willis and Westlund, 1997) is not required, since this tract crosses the cord before ascending and thus is disrupted by the C2 hemisection. It is not clear whether the lower threshold required to activate the diaphragm at 2 months versus 1 day after injury might be due to changes in the contribution of different afferents at chronic stages or changes in the downstream circuitry leading to diaphragm activation. Future experiments in which specific afferents or pathways are activated and/or blocked could help identify the contribution of each pathway to activation of the diaphragm by hindlimb afferent stimulation.

The majority of people living with spinal cord injury are in chronic stages of disease. Thus, we assessed whether limb afferent stimulation could impact diaphragm activity 2 months after injury, which is considered a chronic time point for rodents (Bradbury and Burnside, 2019; Hu et al., 2010; Kjell and Olson, 2016; Salazar et al., 2010; Shibata et al., 2021; Willis and Westlund, 1997). Surprisingly, we found that significant activation of the diaphragm could be achieved at even lower thresholds in chronic compared to acutely injured animals. This result suggests that plasticity within spinal and/or brain circuits can strengthen the pathways activated by limb afferent stimulation and/or their connections to phrenic motor neurons. Further, this indicates that therapies to improve breathing via activation of limb afferents could potentially be even more effective in chronically injured patients. However, it is important to note that C2Hx mice are freely breathing and thus these results may not be applicable to people that are currently on ventilator support. A further limitation of this study is that animals were only provided stimulation at one time point. Future studies should investigate whether repeated stimulation over days or weeks could improve respiratory function beyond what can be achieved by a single application of stimulation.

Our study highlights several factors that could limit the feasibility of using limb afferent stimulation to improve breathing in people with spinal cord injury. First, the current thresholds required to achieve a significant increase in diaphragm activity are 5–10 times that required to elicit limb movement. Other stimulation protocols may yield more effective recovery of respiratory activity; for example: stochastic stimulation has been shown to be more effective than fixed width stimulation to facilitate swallowing after SCI (Kitamura et al., 2024). Another limitation of these studies is they were performed under anesthesia. Responses of respiratory, motor and sensory pathways could differ in non-anesthetized mice. For example, the current required to improve diaphragm function in non-anesthetized animals could be significantly lower. Alternatively, animals might experience adverse behavioral outcomes such as pain responses at the current thresholds required to elicit diaphragm activity. If stimulation acts through pain pathways, then translating it to humans would require anesthesia/analgesia during stimulation, which may not be practical (Phadke et al., 2019; Soriano et al., 2022). Our study did not test for off-target effects, such as altered autonomic reflexes, spasticity, or pain-related behaviors that could result from stimulation, particularly repeated episodes of stimulation (maladaptive plasticity). Thus, a potential increase in the risk of autonomic dysreflexia, cardiovascular dysfunction or chronic pain would have to be considered before performing analogous studies in humans with spinal cord injury. Finally, our study used freely breathing animals in which half of the ascending and descending axons between the brain and spinal cord were intact. Most people with spinal cord injury and respiratory deficiency have less well-defined injuries and thus limb afferent stimulation may not work for all patients. Additional research in animal models on which pathways are essential for activation of breathing by limb afferent stimulation could help to assess which patients might be best suited for this type of therapy.

Our results prompt further examination of alternative methods to restore respiratory function by activating locomotor circuits. For example, we did not assess whether rhythmic, alternating stimulation of left and right sciatic nerves (akin to left–right limb alternation during locomotion) could activate the diaphragm at lower thresholds than stimulation on just one side. Further, we did not test whether stimulation of alternative nerves, such as forelimb or sacral nerves, might be more effective at restoring diaphragm function. Sacral nerve stimulation robustly activates locomotor circuits in neonatal spinal cord preparations (Bonnot et al., 2002; Gordon et al., 2008). Our study also did not test whether passive limb movements could provide sensory afferent stimulation sufficient to improve breathing. Passive leg movements have been shown to be safe and may improve cardiovascular and musculoskeletal outcomes in people with spinal cord injury (Phadke et al., 2019; Soriano et al., 2022), but their impact on recovery of breathing has not been reported. Our study also indicates that epidural (Lorach et al., 2023; Rejc et al., 2017; Rowald et al., 2022; Wagner et al., 2018) or transcutaneous (Ievins and Moritz, 2017; Inanici et al., 2021; Moritz et al, 2024) stimulation of locomotor circuits could have benefits on respiratory function in individuals with severe cervical spinal cord injuries since these methods also activate limb afferents. Individuals with respiratory deficits are not usually included in stimulation trials, which typically focus on recovery of movement.

In conclusion, this study provides the first evidence that limb afferent stimulation can activate a previously paralyzed diaphragm following a C2Hx injury at both acute and chronic stages of injury. Further, we describe an experimental model to investigate the neural pathways by which limb afferents can increase ventilation in uninjured and injured animals. Our results indicate that therapies targeting limb afferents could potentially be used to improve breathing in patients with cervical spinal cord injury.

## Data Availability

The raw data supporting the conclusions of this article will be made available by the authors, without undue reservation.
